# Carbonic anhydrase 8 regulates basophil activation and interleukin 4 production

**DOI:** 10.3389/fimmu.2025.1694105

**Published:** 2025-11-27

**Authors:** Jianya Peng, Chandler B. Sy, Siyuan Zhao, Arman Sawhney, Vanessa Espinosa, Marissa N. Schroeter, John J. Ponessa, Krupa Chavan, Alexander D. Lemenze, Amariliz Rivera, Tibor Rohacs, Mark C. Siracusa

**Affiliations:** 1 Center for Immunity and Inflammation, Rutgers, The State University of New Jersey, Newark, NJ, United States; 2 Department of Medicine, Rutgers, The State University of New Jersey, Newark, NJ, United States; 3 NemaGen Discoveries, Princeton, NJ, United States; 4 Department of Pharmacology, Physiology, and Neuroscience, Rutgers. The State University of New Jersey, Newark, NJ, United States; 5 Department of Pediatrics, Rutgers, The State University of New Jersey, Newark, NJ, United States; 6 Department of Pathology, Immunology and Laboratory Medicine, Rutgers, The State University of New Jersey, Newark, NJ, United States

**Keywords:** carbonic anhydrase 8, basophils, interleukin 4, atopic dermatitis, calcium homeostasis

## Abstract

Carbonic anhydrase (Car) enzymes are a family of metalloenzymes that are traditionally known for their ability to regulate pH and CO_2_ homeostasis. However, emerging studies now demonstrate that Car family members exhibit lineage-specific expression patterns within immune cells. Moreover, it has been shown that genetically and pharmacologically targeting specific Car family members is sufficient to regulate immune cell development and activation. This work has identified Car enzymes as viable therapeutic targets that can influence immunity and inflammation. Here we contribute to this growing body of literature and demonstrate that Car8 is highly expressed by basophils and basophil precursor cells compared to other Car family members. While deletion of Car8 had no effect on basophil development or recruitment, mice deficient in Car8 were protected from basophil- and interleukin (IL)-4-dependent atopic dermatitis-like inflammation. Consistent with these findings, Car8-deficient basophils exhibit defects in the cytokine-stimulated release of IL-4 that is associated with altered calcium signaling pathways. Collectively, these studies reveal the lineage-specific expression patterns of Car8 and its unappreciated function in regulating basophil activation.

## Introduction

Carbonic anhydrase (Car) enzymes are an ancient family of metalloenzymes that have independently evolved in prokaryotes, plants, and mammals (1). While Car enzymes are best known for their classical roles in catalyzing the reversible conversion of carbon dioxide (CO_2_) and water to bicarbonate (HCO_3_^-^) and protons, their convergent evolution across several distinct forms of life suggests they are highly important for the fundamental survival and functioning of an organism (2–5). A total of 16 Car enzyme isoforms have been identified in mammals, among which 13 isozymes (Car 1, 2, 3, 4, 5a, 5b, 6, 7, 9, 12, 13, 14, and 15) are catalytically active. The remaining three members of the family (Car8, Car10, and Car11) lack enzymatic activity due to the absence of the Zn^2+^ active site (6), suggesting their functions may differ from other family members.

Recent studies utilizing mouse models have begun to investigate the functions of Car enzymes and have defined their unique contributions in the context of epilepsy, cancer, glaucoma, and metabolic deficiencies (7–9). Further, emerging studies have also revealed that Car enzymes exhibit lineage-specific expression patterns and regulate immune cell development and activation (10–13). Included among these studies, we previously reported that Car1 is uniquely expressed by mast cell progenitors and can be targeted therapeutically to prevent mast cell responses and mast cell-mediated inflammation (10, 11). This work provoked the hypothesis that Car enzyme family members may also regulate the development and activation of other granulocyte populations.

Similar to mast cells, basophils are a rare granulocyte population that express the high affinity immunoglobulin E (IgE) receptor (FceRIa), secrete histamine, and are potent effector cells (14). Basophil populations rapidly expand and enter inflamed tissues in response to type 2 cytokine responses and play important roles in mediating immunity to parasites and promoting allergic inflammation and pruritic skin disease (15–19). Upon activation, basophils release large amounts of potent effector molecules, including interleukin (IL)-4 and IL-6, which can both initiate and amplify various forms of inflammation.

Here we investigated the expression pattern of Car enzymes in basophils and basophil progenitor cells. Our studies revealed that basophils and their progenitors express Car8, a metabolically inactive Car enzyme family member. While Car8 was not required for basophil development or recruitment, mice deficient in Car8 were protected from basophil-dependent atopic dermatitis (AD)-like disease. Furthermore, Car8-deficient basophils were found to have altered calcium signaling pathways that were associated with a defect in their release of IL-4 and IL-6 upon cytokine stimulation. These studies provide substantial insight into the factors that regulate basophil activation and identify previously unappreciated functions for Car8, a metabolically inactive member of the carbonic anhydrase family of enzymes.

## Results

**Figure 1:**
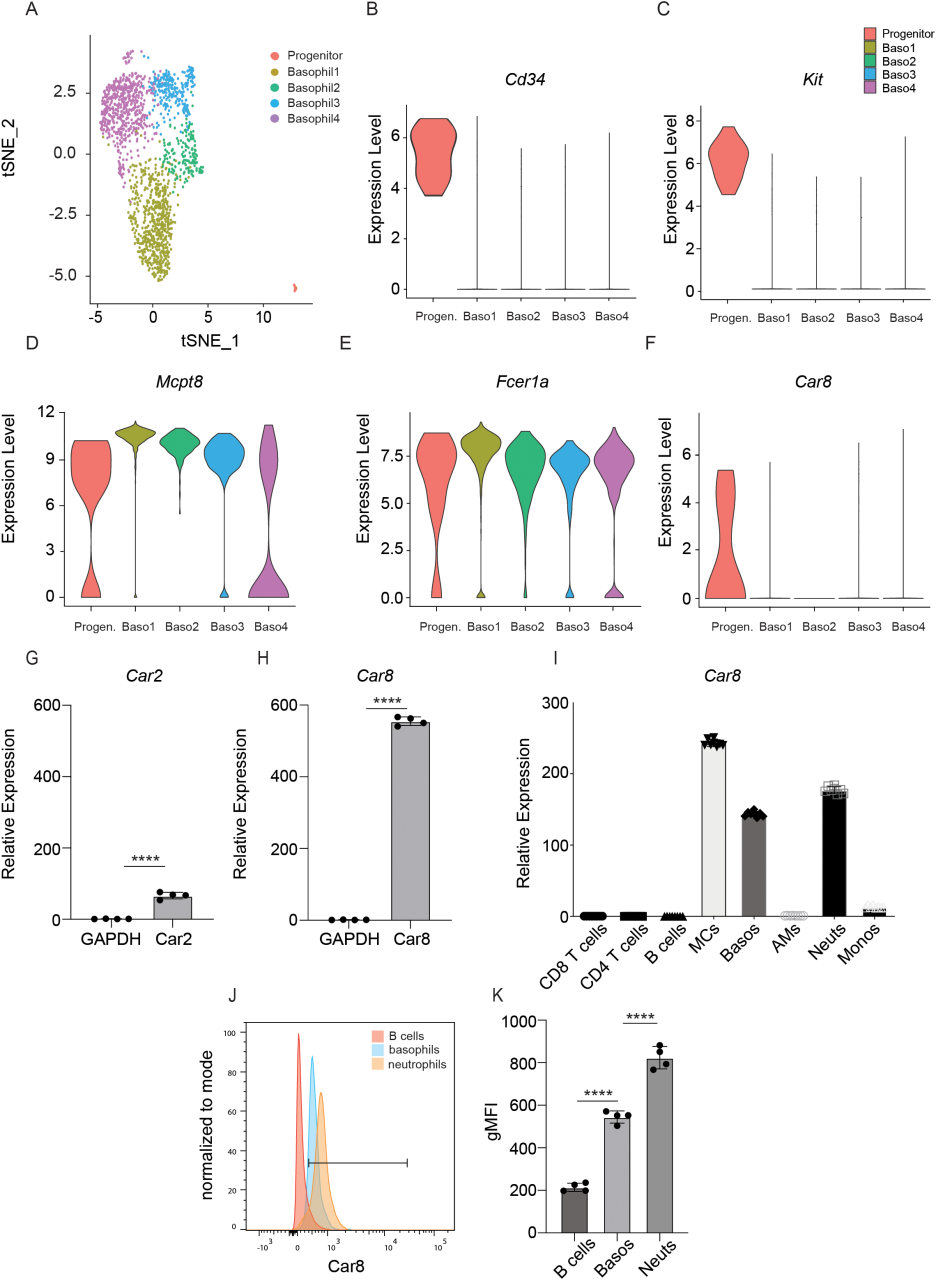
**(A)**, Single cell RNA-seq of *Mcpt8*-expressing basophils and basophil progenitors was evaluated (GEO accession number: GSE206589). Expression of **(B)***CD34*, **(C)***c-Kit*, **(D)***Mcpt8*, **(E)***Fcer1a*, and **(F)***Car8* were evaluated in the 4 unique clusters identified by t-distributed stochastic neighbor embedding analysis. **(G, H)**, RNA from sort-purified FceRIa^+^, CD49b^+^, c-Kit^-^ bone marrow-derived basophils and other cell types were extracted and expressions of *Car2* and *Car8* in basophils were evaluated via RT-qPCR and normalized to their own *Gapdh*. **(I)** Expressions of *Car8* in splenic basophils and other immune cell types were evaluated via RT-qPCR and normalized to alveolar macrophages *Gapdh*. **(J, K)**, Intracellular staining of Car8 from the splenic cells of wild type (WT) mice, with geometric MFI quantified. **(G–K)**, Individual experiments were repeated 3–5 times with 3–4 biological replicates per group. Statistical analysis between two indicated groups was performed using Student’s t-test. ****, p < 0.0001. Error bars represent SD.

Our previous work revealed that the Car1 is a unique and identifying feature of mast cell progenitors and demonstrated that Car1 could be targeted without influencing basophil populations (10, 11). Given emerging data suggesting that Car enzyme family members have lineage-specific expression patterns within immune cells, we sought to determine whether specific Car family members were associated with basophils and/or their progenitors. To test this, we accessed publicly available single cell RNA-seq data that was generated from mast cell protease 8 (Mcpt8) green fluorescent protein (GFP) reporter mice, as Mcpt8 is a basophil-specific protease (20). Single cell RNA-seq was performed on Mcpt8-expressing cells sort-purified from the bone marrow and spleen (20). Marker gene analysis allowed us to identify four distinct basophil populations that were defined by their expression of *Fcer1a* and *Mcpt8* (named Baso1, 2, 3, and 4) (**Figures 1A-E**). Additionally, a basophil progenitor population (Progenitor) was also identified by its shared expression of *Fcer1a* and *Mcpt8* along with the progenitor cell markers *Cd34* and *Kit* (**Figures 1A-E**). We next evaluated whether these distinct cell clusters expressed Car family members. Broad analysis of Car enzyme expression revealed that expression of only two family members were detected in the various cell populations (**Supplementary Figure 1**). *Car2* was expressed by the Baso2 population and the basophil progenitor cells, while *Car8* was expressed by basophil progenitor cells (**Figure 1F**, **Supplementary Figure 1**). To better evaluate the expression of *Car2* and *Car8* within mature basophils and without the possible limitations of the Mcpt8 reporter system, we sort-purified FcERIa^+^, CD49b^+^, c-Kit^-^ bone marrow-derived basophils and performed RT-PCR analysis. Interestingly, when normalized to *Gapdh*, *Car8* expression was found to be expressed more robustly than *Car2* in mature bone marrow-derived basophils (**Figures 1G, H**). These data further contribute to a growing body of literature demonstrating the unique expression patterns of Car enzymes within immune cells. Moreover, the robust expression of *Car8* by basophils suggests it may regulate their development and/or function. To gain a better understanding of *Car8* expression patterns within a boarder set of immune cells, we sort-purified T cells, B cells, mast cells, basophils, alveolar macrophages, neutrophils, and monocytes and performed RT-PCR analysis. Interestingly, *Car8* was found to be highly expressed by mast cell, splenic basophils, and neutrophils, but not the other immune cells evaluated (**Figure 1I**). Next, we sought to evaluate how Car8 protein levels correlated with our transcriptional analysis. To test this, we perform intracellular staining for Car8 protein. Consistent with transcriptional studies, Car8 protein was not detected in B cells, but was expressed by splenic mature basophils and neutrophils (**Figures 1J, K**). These data demonstrate that *Car8* is expressed during basophil development and is present within mature basophils. Moreover, the unique expression of *Car8* within granulocyte populations further provokes the hypothesis that it may regulate the development and/or activation of these highly specialized innate immune cell populations.

**Figure 2:**
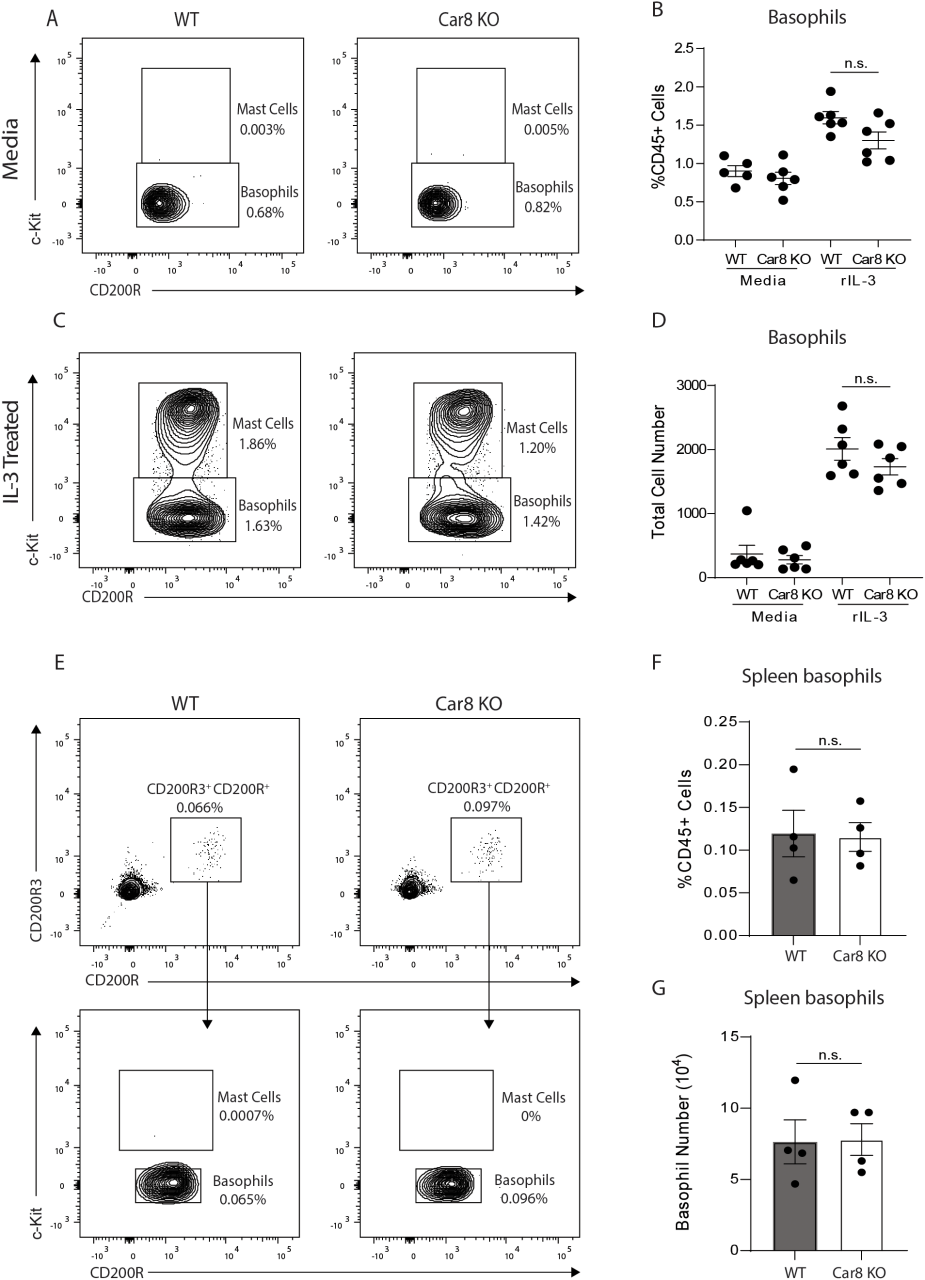
**(A–D)**, Bone marrow-resident progenitor cells from WT or Car8-deficient mice were cultured with media or IL-3 and CD200R^+^ c-Kit^+^ mast cells and CD200R^+^, c-Kit^-^ basophils were quantified. **(E–G)**, CD200R3^+^, CD200R^+^, c-Kit^-^ basophils and CD200R3^+^, CD200R^+^, c-Kit^+^ mast cells were quantified from the spleens of WT or Car8-deficient mice at baseline. **(A–G)** Individual experiments were repeated 3–5 times with at least 3 biological replicates per group. Statistical analysis was between two indicated groups was performed using Student’s t-test. *, p < 0.05; **, p < 0.01; ***, p < 0.001; ****, p < 0.0001; n.s., not significant. Error bars represent SD.

In our studies, we chose to further investigate whether Car8 plays a role in regulating basophil responses. First, we sought to determine whether Car8 regulates basophil development by isolating bone marrow cells from the femurs of wild type (WT) and Car8-deficient mice and culturing them in the presence of IL-3. Compared to media-treated controls, WT mice showed significant increases in the percentage and total number of CD200R^+^, c-Kit^-^ basophils and CD200R^+^, c-Kit^+^ mast cells post-culture (**Figures 2A–D**). Interestingly, Car8-deficient bone marrow cultures exhibited equivalent increases in basophils and mast cells following IL-3 treatment compared to cultures derived from WT mice (**Figures 2A–D**), suggesting that Car8 is not required for basophil development from bone marrow progenitor cells. Additionally, when CD200R3^+^, CD200R^+^, c-Kit^-^ basophil populations were evaluated in the spleens of WT and Car8-deficient mice, there was no significant difference in percentage or total number (**Figures 2E–G**). These data further suggest that basophil populations develop normally in the absence of Car8.

**Figure 3:**
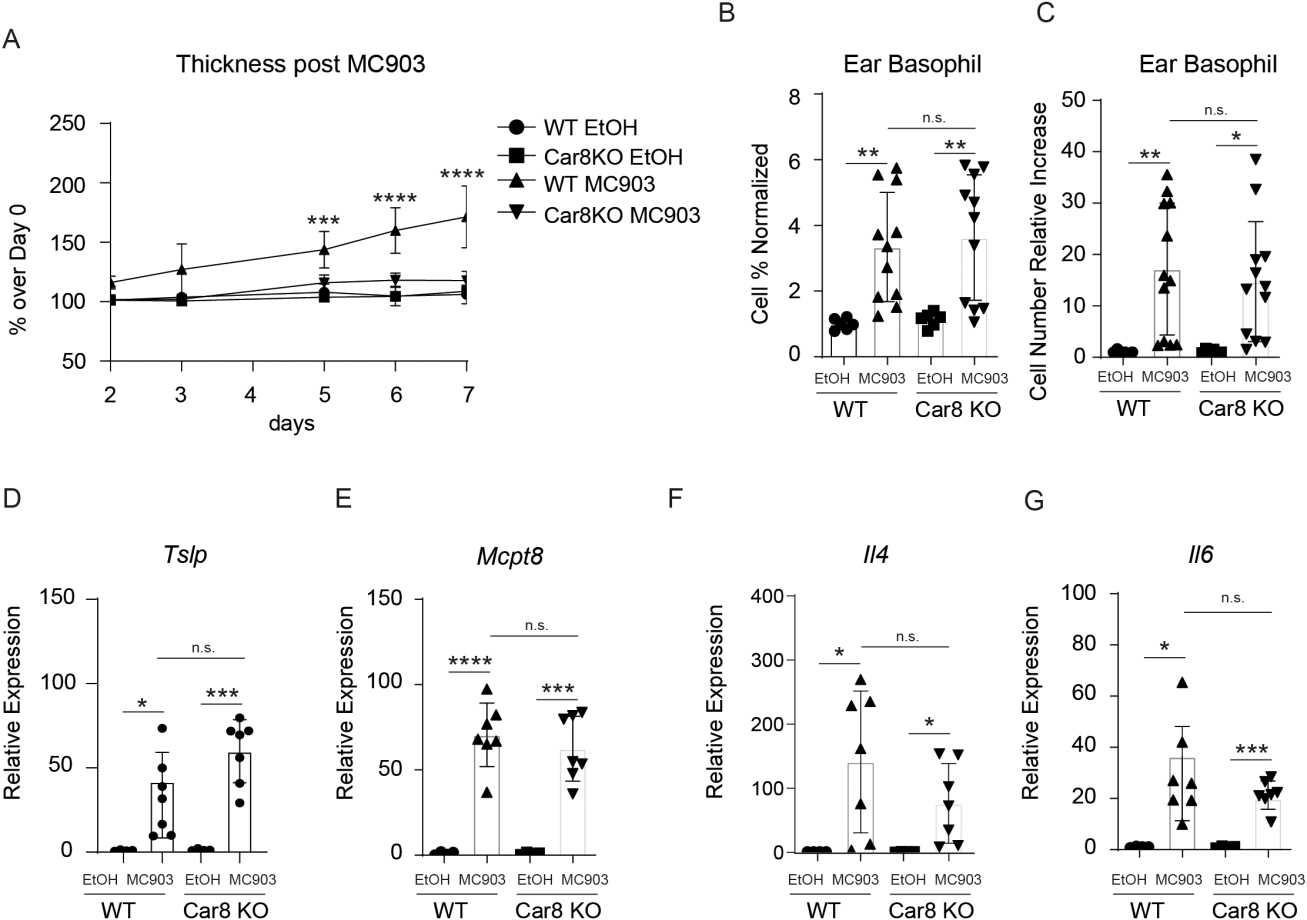
The ears of WT or Car8-deficient mice were treated with ethanol (EtOH) or MC903 and **(A)** ear thickness was measured. **(B, C)**, Following treatment with ethanol or MC903 cells were isolated from the ears and basophil populations were quantified. Expression levels of **(D)***Tslp*, **(E)***Mcpt8*, **(F)***Il4* and **(G)***Il6* were evaluated in the ears of mice following treatment with EtOH or MC903. **(A–G)** Individual experiments were repeated 5 times with at least 3 biological replicates per group. Statistical analysis between two indicated groups was performed using Student’s t-test. *, p < 0.05; **, p < 0.01; ***, p < 0.001; ****, p < 0.0001; n.s., not significant. Error bars represent SD.

The studies presented above suggest that Car8 is expressed during basophil development but is not required for their lineage commitment. This provoked that hypothesis that Car8 may be required for proper basophil migration and/or activation. To better evaluate this, we employed a basophil-dependent model of atopic dermatitis (AD)-like disease (21). In this model, topical treatment of skin with the irritant MC903, a low-calcemic analog of vitamin D3, results in production of cytokine alarmins by keratinocytes and the rapid recruitment of basophils to the skin. These skin-resident basophils produce robust amounts of IL-4 and cooperate with group 2 innate lymphoid cells (ILC2s) to promote skin inflammation (21–23). Critically, targeting basophils or IL-4 following MC903 treatment results in significantly reduced skin inflammation, thereby providing an ideal system to evaluate basophil responses *in vivo* (21). As expected, treatment of WT mice with MC903 resulted in marked skin inflammation as indicated by significantly increased ear skin thickness compared to vehicle-treated controls (**Figure 3A**). In contrast, Car8-deficient mice were protected from MC903-induced inflammation and exhibited reduced skin thickness compared to controls (**Figure 3A**). Histological analysis of skin sections revealed that MC903-treated WT mice exhibited substantially increased epidermal hyperplasia and cellularity of the dermis, as indicated by significantly increased numbers of skin-resident CD45^+^ cells (**Supplementary Figure 2A, B**). In contrast, Car8-deficient mice treated with MC903 exhibited substantially less epidermal hyperplasia and significantly reduced cellularity (**Supplementary Figure 2A, B**). These data provoked the hypothesis that basophil responses might be reduced in mice lacking expression of *Car8*. To better evaluate this, we isolated skin-resident cells and performed flow cytometric analysis to identify basophils. Consistent with published studies, MC903 treatment of WT mice resulted in significant increases of FceRIa^+^, CD200R3^+^, CD200R^+^, c-Kit^-^ skin-resident basophils populations by both percentage and total number (**Figures 3B, C**). Interestingly, significantly increased percentages and total numbers of basophils were also observed in the skin of Car8-deficient mice treated with MC903 (**Figures 3B, C**). Additional transcriptional analysis revealed that the WT and Car8-deficient animals also exhibited equivalent increases in the expression of the basophil-promoting cytokine alarmin *Tslp*, the basophil-specific protease *Mcpt8*, and the basophil-derived effector molecules *Il4* and *Il6* (**Figures 3D–G**). These studies demonstrate that Car8-deficient mice have intact MC903-induced basophil responses.

**Figure 4:**
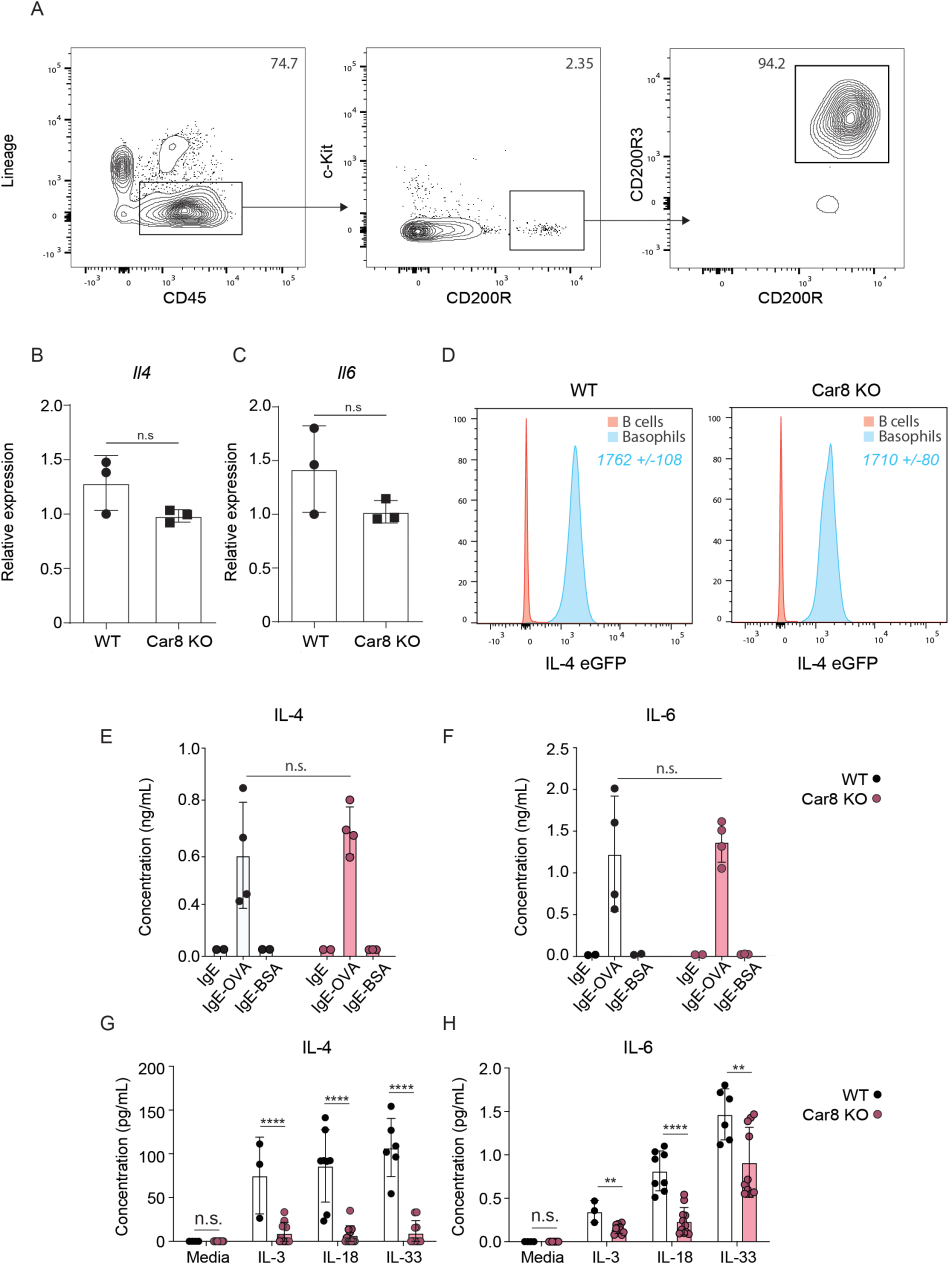
**(A)** Lin^-^, CD45^+^, c-Kit^-^, CD200R^+^, CD200R3^+^ basophils were sort-purified from the spleens of WT or Car8-deficient mice treated with IL-3-anti-IL-3 complexes (IL-3C). Expressions of **(B)***Il4* and **(C)***Il6* from sort-purified splenic basophils were evaluated via RT-qPCR and normalized to IL-3C stimulated WT-derived basophils. **(D)** IL-4 eGFP expressions in Lin^-^, CD45^+^, c-Kit^-^ CD200R^+^, CD200R3^+^ splenic basophils were isolated and evaluated from IL-4Get Car8-sufficient and IL-4Get Car8-deficient mice. **(E, F)** Lin^-^, CD45^+^, c-Kit^-^, CD200R^+^, CD200R3^+^ basophils were sort-purified from the spleens of WT or Car8-deficient mice, and equal numbers of each group were treated with OVA-specific IgE and followed by BSA or OVA stimulation overnight. IL-4 and IL-6 protein levels were evaluated in cell-free supernatants post-culture by ELISA. **(G, H)**, Lin^-^, CD45^+^, c-Kit^-^ CD200R^+^, CD200R3^+^ basophils were sort-purified from the spleens of WT or Car8-deficient mice treated with IL-3-anti-IL-3 complexes and cultured in the presence of media, IL-3, IL-18, or IL-33. IL-4 and IL-6 protein levels were evaluated in cell-free supernatants post-culture by ELISA. **(A–H)** Individual experiments were repeated 3–5 times with at least 3 biological replicates per group. Statistical analysis between two indicated groups was performed using Student’s t-test. **, p < 0.01; ****, p < 0.0001; n.s., not significant. Error bars represent SD.

The data presented above demonstrate that Car8-deficient mice have significantly reduced skin inflammation in a model of basophil-dependent AD-like disease despite having equivalent levels of basophils migrating to the skin. These data provoked the hypothesis that Car8-deficient basophils may have altered effector functions. To evaluate this, we injected WT and Car8-deficient mice with IL-3-anti-IL-3 complexes (IL-3C), a treatment that promotes systemic basophilia, and sort-purified splenic basophils (**Figure 4A**). We first evaluated whether WT and Car8-deficient basophils exhibited equivalent transcriptional levels of *Il4* and *Il6*. Consistent with the presence of *Il4* and *Il6* expressions in the skin of both WT and Car8-deficient mice post-MC903 treatment, no significant differences in *Il4* or *Il6* transcripts were observed between the two groups (**Figures 4B, C**). We then crossed the IL-4-eGFP transcriptional reporter mouse (4get mice) onto a Car8-deficent background (24). Corroborating our transcriptional studies, WT and Car8-deficient basophils exhibited equivalent levels of IL-4-eGFP by flow cytometric analysis (**Figure 4D**). Given that MC903-induced inflammation is highly dependent on IL-4 production by basophils, we sought to determine if the release of IL-4 protein differed in the absence of Car8. To test this, we sort-purified equal numbers of WT and Car8-deficient basophils, loaded them with ovalbumin (OVA)-specific IgE, and stimulated them with OVA or bovine serum albumin (BSA) as a negative control. As expected, IL-4 and IL-6 levels were found at higher levels in the cell-free supernatants of WT basophils stimulated with IgE and OVA compared to controls (**Figures 4E, F**). Interestingly, culture supernatants from Car8-deficient basophils stimulated with IgE and OVA exhibited equivalent levels of IL-4 and IL-6 to those of WT controls. These data demonstrate the WT and Car8-deficient basophils respond similarly following IgE-mediated activation.

Studies have demonstrated that the IgE-mediated activation of basophils results in a rapid activation pathway that is associated with degranulation (25). In contrast, the activation of basophils following cytokine stimulation is reported to be more gradual and involves distinct singling pathways (26). Given that the MC903 model of AD-like diseases is not antigen driven and highly-dependent on cytokine alarmins (21, 23), we next evaluated whether WT and Car8-deficient basophils differ in their activation following cytokine stimulation. To test this, basophils were sort-purified from WT or Car8-deficient mice following IL-3C treatment and cultured in the presence or absence of the basophil activating cytokines IL-3, IL-18, or IL-33. Following cytokine treatment, IL-4 and IL-6 were measured in cell-free supernatants by ELISA. As expected, supernatants from WT basophils stimulated with IL-3, IL-18, or IL-33 exhibited significantly increased levels of IL-4 and IL-6 compared to media treated controls (**Figures 4G, H**). In contrast, Car8-deficient basophils stimulated with the same cytokines showed significantly reduced levels of IL-4 and IL-6 releases into the cell-free supernatants. These data strongly suggest that Car8 is required for optimal cytokine production by basophils following cytokine stimulation (**Figures 4G, H**).

**Figure 5:**
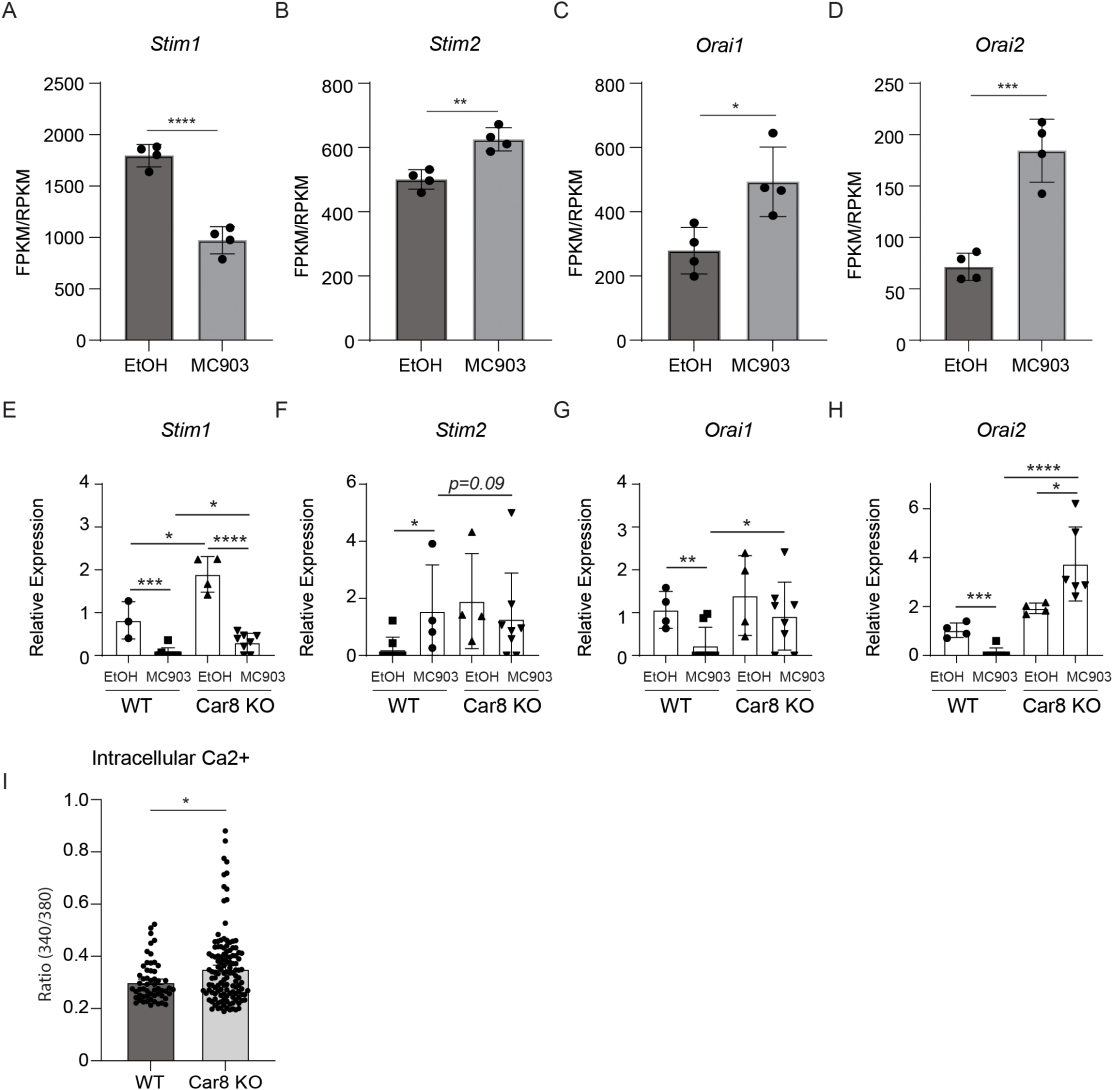
The ears of WT mice were treated with ethanol (EtOH) or MC903 and RNA-seq was performed. **(A-D)**, Expression levels ofthe calcium-associated molecules *Stim1*, *Stim2*, *Orai1*, and *Orai2* were evaluated (fragments per kilobase million (FPKM)/reads per kilobase million (RPKM)). **(E-H)**, The ears of WT or Car8-deficient mice were treated with EtOH or MC903 and *Stim1*, *Stim2*, *Orai1*, and *Orai2* were evaluated by RT-PCR. **(I)**, Lin^-^, CD45^+^, c-Kit^-^, CD200R^+^, CD200R3^+^ basophils were sort-purified from the spleens of WT or Car8-deficient mice treated with IL-3-anti-IL-3 complexes and Ca2^+^ imaging measurements were performed. **(A-H)** Individual experiments were repeated 3–5 times with at least 3 biological replicates per group. Statistical analysis between two indicated groups was performed using Student’s t-test. *, p < 0.05; **, p < 0.01; ***, p < 0.001; ****, p < 0.0001. Error bars represent SD.

The data presented above suggest that Car8 regulates the release of IL-4 and IL-6 from basophils in response to cytokine-mediated activation. It is well established that the release of cytokines by activated basophils is mediated by changes in cytoplasmic calcium levels (27, 28). A primary calcium regulator in this process is the calcium release-activated calcium (CRAC) channel that opens to allow calcium into a cell once calcium stores have been depleted (29). Upon cellular activation, inositol 1,4,5 trisphosphate (IP_3_) engages the inositol 1,4,5 trisphosphate receptor (IP_3_R) to promote the release of calcium from the endoplasmic reticulum (ER) as the intracellular source to increase cytoplasmic calcium level (30, 31). Previous studies have shown that the stromal interaction molecules (Stim) 1 and 2 (calcium sensors that reside in the endoplasmic reticulum) will then activate and operate with Orai1 and 2 (components of the CRAC channel at the cell membrane) to allow calcium to enter the cell and thereby restore calcium levels (26, 32–35). More recent studies have shown that Car8 acts as an allosteric inhibitor of IP_3_R, and Stim1 and Stim2 are differentially required for basophil responses (26, 36–38). Therefore, we first sought to determine whether these pathways are altered in the context of MC903-induced AD-like disease. To do this, we analyzed publically available RNA-seq data performed on control and MC903 inflamed skin (39). While *Stim1* expression was significantly reduced following MC903 treatment, *Stim2*, *Orai1* and *Orai2* were all significantly increased in response to MC903 (**Figures 5A–D**). These data demonstrate that these pathways are altered in the context of AD-like disease. Next, we sought to determine if these molecules were dysregulated in the absence of Car8. Interestingly, *Stim1* and *Sim2* expressions were significantly increased in the absence of Car8 at baseline (**Figures 5E, F**). *Stim1* expression post-MC903 treatment was similarly decreased in both WT and Car8-deficient basophils. However, *Stim2* expression was not significantly increased in Car8-deficient mice following MC903 treatment unlike in treated WT mice (**Figures 5E, F**). Moreover, *Orai1* and *Orai2* expressions were also significantly increased post-MC903 treatment in Car8-deficient mice compared to WT controls (**Figures 5G, H**). Collectively, these findings suggest that a lack of Car8 may lead to altered STIM and ORAI pathways and dysregulated calcium gradient. While these data provoke the hypothesis that Car8 may regulate calcium response in basophils, the changes observed in this global knockout model may not be basophil-specific. Therefore, to directly evaluate whether Car8 regulates calcium responses in basophils, we sort-purified basophils from WT and Car8-deficient mice and performed calcium imaging on the cells. Consistent with dysregulated calcium gradient, Car8-deficient basophils exhibited higher baseline calcium levels than WT control basophils (**Figure 5I**). Critically, Yoshikawa et al. showed that production of IL-4 from WT bone marrow-derived basophils could be completely inhibited by extracellular or intracellular Ca^2+^ chelators, suggesting that properly maintaining the Ca2^+^ gradient at baseline is required for IL-4 production (26). Similarly, these studies suggest that Car8 is required for basophils to maintain calcium homeostasis and gradient levels that are required for basophils to release IL-4 and contribute to IL-4-mediated inflammation.

## Discussion

Carbonic anhydrase enzymes represent a prime example of convergent evolution via their independent development in various classes of life (5). Interestingly, our work and that of others are beginning to reveal that the alpha class of isozymes found in mammals exhibit extremely distinct expression patterns in various tissues and immune cells (10, 11). Moreover, emerging studies are also defining the unique roles these specific isozymes play in the context of both immune cell development and function, indicating that they may possess important therapeutic potential. Among these isozymes, Car8 represents one of only three family members that are known to be metabolically inactive, suggesting it has evolved functions beyond the ability to regulate pH and CO_2_ homeostasis.

Here we demonstrate that basophil precursor cells uniquely express high levels of *Car8* compared with other Car enzyme family members. While rare in number compared to other leukocyte populations, basophils are potent effector cells that play established roles in the context of parasitic immunity, allergic inflammation, and pruritic disease (16, 40–42). Additionally, emerging studies have also revealed the important contributions of basophils to lung development, kidney fibrosis, auto immune disorders, and even anti-tumor immunity (16, 42–45). The diverse functions of basophils are perhaps not surprising given their ability to rapidly migrate to tissues and activate in response to numerous signals including IgE crosslinking, protease actively, and cytokine stimulation (16, 41, 42, 45). Following exposure to these stimuli, basophils produce a vast array of effector cytokines including robust levels of IL-4 and IL-6 that are known to mediate many of their potent effects.

While the data presented here show that Car8 is not required for basophil development or migration, our work reveals that Car8 is needed for their optimal release of basophil-derived IL-4 and IL-6 following cytokine stimulation. Consistent with defects in IL-4 production, Car8-deficient animals were protected from basophil- and IL-4-dependent AD-like disease. To the best of our knowledge these studies are the first to demonstrate the expression and function of Car8 in basophils and its ability to regulate basophil-dependent inflammation. Further, these studies contribute to an increasing body of literature illustrating the importance of Car enzyme family members in regulating immune responses.

It is well established that Car8 is highly expressed by dorsal root ganglion neurons where it operates as an inhibitor of inositol trisphosphate receptor-1 (ITPR1) and regulates intracellular calcium levels (36, 46). Given the known role that intracellular calcium plays in regulating the release of IL-4 by basophils, we hypothesized that Car8-deficient basophils may exhibit dysregulated calcium responses. Consistent with this hypothesis Car8-deficient basophils showed significantly increased intracellular calcium levels compared to controls. Previous work in other cell types has demonstrated that sustained elevations in intracellular calcium can suppress signaling pathways and make cells less responsive to additional stimulation and thereby inhibit the optimal release of effector molecules (47–50). The work presented here is consistent with those reports and suggests that the tightly regulated calcium levels are required for basophils to properly respond to cytokine stimulation. While the data here identify Car8 as an important regulator of these processes, additional work is required to determine the exact calcium channels and/or pumps that are directly or indirectly affected by Car8. It is also possible that Car8 may regulate post-translational modifications (PTMs) targeting these calcium regulators, as a substantial body of literature over the past 15 years has demonstrated that these critical molecules and channels are regulated through PTMs in various cell types, including immune cells (51–53). Studies of this nature are especially important given that Car8 does not appear to affect the antibody-mediated activation of basophils which is known to operate via distinct calcium signaling pathways than those initiated following cytokine-mediated activation (26). Moreover, it is worth investigating whether Car8 similarly regulates calcium responses and activation in mast cells and neutrophils, which also express high levels of Car8. Given the specific expression of Car8 within granulocyte lineages, it is possible that this would be a conserved mechanism of activation across these cell types. It is also important to highlight that deficient activation of other Car8-expressing granulocytes may contribute to the dramatic phenotype observed in the MC903 model, given that the Car8 mouse model is not basophil-specific. Additionally, future work is also needed to determine whether the expression of Car8 in granulocytes is conserved in humans to further evaluate its therapeutic potential.

In summary, these studies reveal the unique expression of Car8 by basophil precursor cells and highlights its previously unappreciated ability to regulate important aspects of basophil activation. To the best of our knowledge these studies are the first to investigate the expression of Car8 within immune cells and add to an emerging body of literature identifying the unique expression patterns of Car family members within hematopoietic lineages. Moreover, this work suggests that inactive Car isozymes have also evolved the ability to regulate immunity and inflammation and as such may represent viable therapeutic targets.

## Materials and methods

### Mouse experiments

Eight to ten-week-old female and male wild type C57BL/6J, C57BLKS/J, IL-4/GFP-enhanced transcript (4Get) and Car8 knockout mice were purchased from Jackson or generously provided by Dr. Roy Levitt at the University of Miami Miller School of Medicine (36). Then breeding colonies were established. All mice were maintained in specific pathogen-free facilities at the Rutgers New Jersey Medical School. All protocols were approved by the Rutgers Institutional Animal Care and Use Committee, PROTO202000017.

Mice were treated once a day topically with 2 nmol of MC903 (calcipotriol; Tocris Bioscience) in 20 µl ethanol for 7 days as described previously (21).

### Flow cytometry, intracellular staining & cell sorting

Cells were stained with monoclonal anti-mouse fluorescently conjugated antibodies: CD45R/B220 (clone RA3-6B2); CD3 (clone 145-2C11); CD11b (clone MI/70); CD11c (clone N418); IgE (clone 23G3); FcERIa (clone MAR-1);CD49b (clone DX5); CD45 (clone 30-F11); CD64 (clone X54-5/7.1); CD90 (clone 5E10); CD127 (clone A7R34); CD200R1 (clone OX110); CD200R3 (clone Ba13); EpCAM (clone G8.8); F4/80 (clone BM8); γδTCR (clone eBioGL3); Siglec-F (clone E50-2440); Ly6G (clone 1A8); Ly6C (clone AL-21); IL-5 (clone TRFK5); IL-13 (clone eBio13A); podoplanin (clone 8.1.1); and TER-119 (clone TER-119) from eBioscience (Thermo Fisher Scientific) or BD Biosciences. Basophils were gated and analyzed as CD45^+^CD3^−^CD19^−^NK1.1^−^Ly6G^−^Siglec-F^−^ CD49b^+^CD200R1^+^ CD200R3^+^FcERIa^+^.

Post-surface staining, cells undergoing intracellular staining were placed in fixation buffer (eBioscience) for 10 mins, and then permeabilized in permeabilization buffer (eBioscience) for 1 hour. Cells were incubated with Anti-Mouse Car8, Biotin (Abcam) for 1 hour, and then with secondary antibody attached to Streptavidin for 1 hour.

### Mouse primary cell culture & treatment

Basophils were isolated and sort-purified from bone marrow. Equal numbers of WT and Car8-deficient basophils were then cultured in the presence or absence of the basophil activating cytokines IL-3, IL-18, IL-33; or culture supernatant containing OVA-specific IgE secreted by the hybridoma TOϵ (54) (kindly provided by Dr. Paul Bryce) and then OVA (Sigma Cat.# O1641) and IL-4 and IL-6 were measured in cell-free supernatants by ELISA. ELISA was performed to measure IL-4 (clones 11B11 and BVD6-24G2) and IL-6 (MP5-32C11) levels in the cell-free supernatants.

### Basophil sort & calcium signaling

WT mice were injected intravenously with a combination of 1 µg mouse recombinant IL-3 (cat. no. 403-ML; R&D Systems) and 0.5 μg of anti-IL-3 antibody (clone MP2-8F8; BioLegend) in 200 μl of PBS 3 days before euthanasia as described previously (55, 56). At necropsy, single-cell suspensions of spleens were prepared, and basophil populations were sort-purified as described earlier using a FACSAria II (BD Biosciences). Purity for all cell populations was 98% or greater.

Ca2^+^ imaging measurements were performed with an Olympus IX-51 inverted microscope equipped with a DeltaRAM excitation light source (Photon Technology International, PTI), as described earlier (57). 5x10^5 ~1x10^6 cells were loaded with 1 mM fura-2 AM (Invitrogen) for 40 min before the measurement at 37˚C, and dual-excitation images at 340 and 380 nm excitation wavelengths were detected at 510 nm with a Roper Cool-Snap digital CCD camera. Measurements were conducted in the Mg2^+^ free HBSS, supplemented with 2 mM CaCl_2_. Data analysis was performed using the Image Master software (PTI).

### Quantitative PCR

Total tissue RNA was extracted with Trizol reagent (Invitrogen, San Diego) followed by phenol/chloroform extraction and isopropanol precipitation. RNA from sorted cells was extracted using the RNeasyMinikit (Qiagen) following the manufacturer’s instructions. Complementary DNA was generated per standard protocol with SuperScript Reverse Transcriptase II (cat. no. 18064014; Invitrogen) and used as input for reverse transcription PCR. Reverse transcription PCR data were analyzed using the 2^-ΔΔct^ method with the SYBR Green Chemistry (Thermo Fisher I Scientific), with β-actin (Actb) serving as the endogenous housekeeping gene. All reactions were run on an ABI 7500 Fast Real-Time PCR System (Applied Biosystems). Samples were normalized to vehicle treated controls. The following QuantiTect primer assays from QIAGEN were used: IL-4 (QT00160678); IL-5 (QT00099715); IL-13 (QT00099554); Mcpt8 (QT00131565); TSLP (QT00198261); STIM1 (QT00105119); STIM2 (QT00289009); Orai1 (QT00285775); Orai2 (QT00304738).

### Statistics

Results are shown as the mean ± s.d. P < 0.05 was considered as significantly different. Statistical analysis between two indicated groups was performed using Student’s t-tests in Prism 8 (GraphPad Software).

## Data Availability

The datasets presented in this study can be found in online repositories. The names of the repository/repositories and accession number(s) can be found in the article/**Supplementary Material**.

## Supplementary material

Supplementary Figure 1 Single cell RNA-seq of *Mcpt8*-expressing basophils and basophil progenitors was evaluated (GEO accession number: GSE206589). Expressions of Car enzyme family members were evaluated in the 4 unique basophil clusters.

Supplementary Figure 2 Histopathology/Representative hematoxylin and eosin staining (scale bar 50μm; original magnification, 20X) of ears of EtOH or MC903 treated WT and Car8-deficient mice. CD45^+^ immune infiltrates were evaluated and quantified. Individual experiments were repeated 3–5 times with at least 3 biological replicates per group. Statistical analysis between two indicated groups was performed using Student’s t-test. **, p < 0.01. Error bars represent SD.
