# Supplementary figures and images for: Carbonic anhydrase 8 regulates basophil activation and interleukin 4 production
Source: Front Immunol. 2025 Nov 27;16:1694105. doi: 10.3389/fimmu.2025.1694105 (PMC12695864; doi:10.3389/fimmu.2025.1694105)

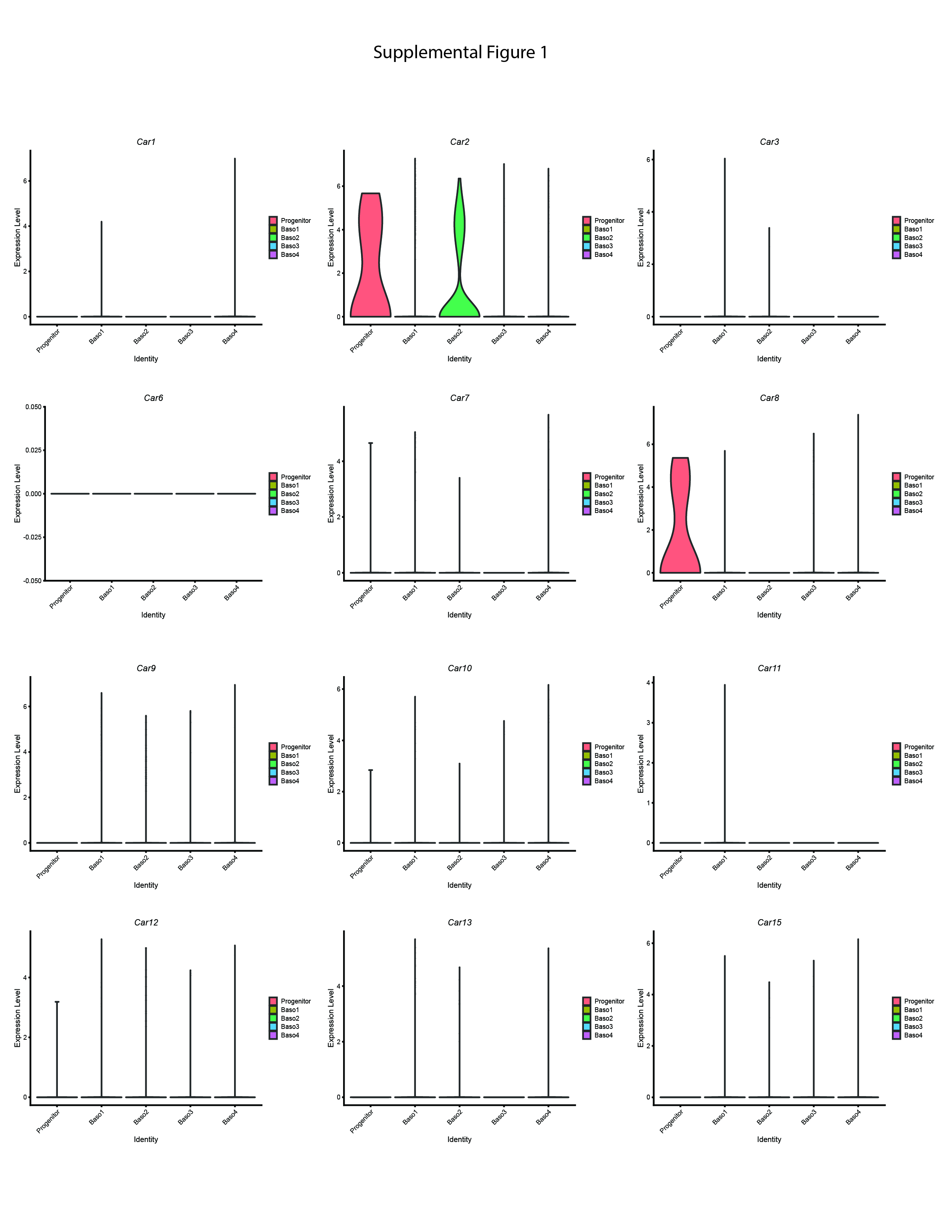

Supplement: Supplementary Figure 1 — Single cell RNA-seq of Mcpt8-expressing basophils and basophil progenitors was evaluated (GEO accession number: GSE206589). Expressions of Car enzyme family members were evaluated in the 4 unique basophil clusters. [file Image1.tif]

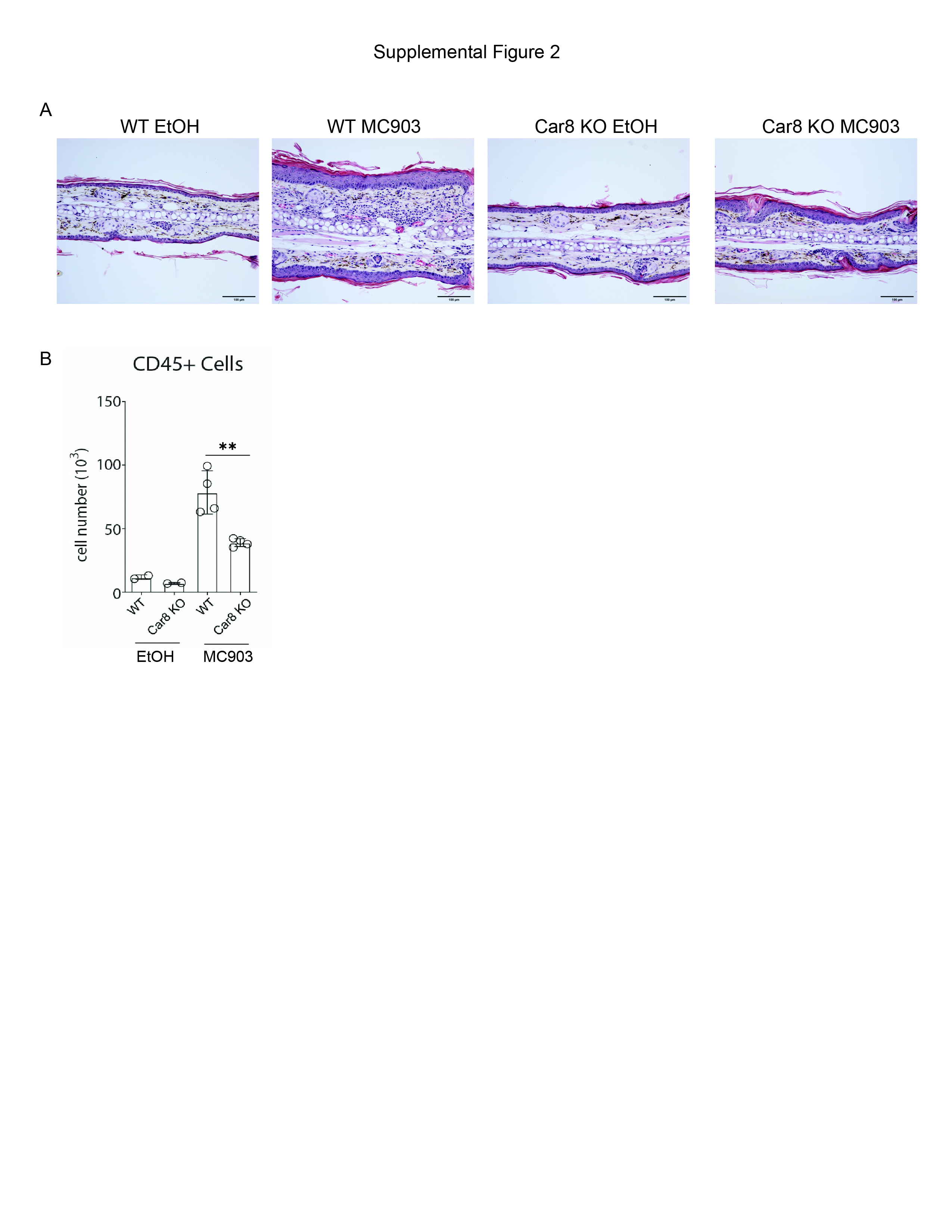

Supplement: Supplementary Figure 2 — Histopathology/Representative hematoxylin and eosin staining (scale bar 50μm; original magnification, 20X) of ears of EtOH or MC903 treated WT and Car8-deficient mice. CD45+ immune infiltrates were evaluated and quantified. Individual experiments were repeated 3–5 times with at least 3 biological replicates per group. Statistical analysis between two indicated groups was performed using Student’s t-test. **, p < 0.01. Error bars represent SD. [file Image2.tif]
